# “Roundabout” Bailout Stenting for Subacute Right Coronary Artery Obstruction After Self-Expandable Transcatheter Aortic Valve Replacement

**DOI:** 10.1016/j.jaccas.2026.107645

**Published:** 2026-03-25

**Authors:** Tatsuki Furusawa, Hiroyuki Kiriyama, Yuji Manabe, Tatsuya Kamon, Shun Minatsuki, Koki Nakanishi, Masahiko Ando, Haruo Yamauchi, Minoru Ono, Norihiko Takeda

**Affiliations:** a Department of Cardiovascular Medicine, The University of Tokyo, Tokyo, Japan; b Department of Cardiovascular Surgery, The University of Tokyo, Tokyo, Japan

**Keywords:** bailout PCI, coronary artery obstruction, self-expandable valve, transcatheter aortic valve replacement

## Abstract

**Background:**

Coronary artery obstruction after transcatheter aortic valve replacement (TAVR) is a rare but life-threatening condition. A bailout strategy has not yet been standardized.

**Case Summary:**

A 91-year-old woman underwent TAVR with a self-expanding valve. Shortly after an initially uneventful procedure, she developed cardiac arrest due to subacute occlusion of the right coronary artery. Although the right coronary sinus was sequestered by the transcatheter heart valve, selective right coronary artery access was achieved via a “roundabout” approach from the noncoronary cusp. Two overlapping zotarolimus-eluting stents across the native commissure restored coronary flow, with satisfactory midterm patency.

**Discussion:**

The mechanisms of coronary artery obstruction and the spatial relationship between the native valve and transcatheter heart valve vary according to patient anatomy and device configuration, necessitating tailored bailout strategies after TAVR.

**Take-Home Message:**

A “roundabout” approach via the adjacent cusp may allow coronary access and revascularization when the coronary sinus is sequestered.

## Visual Summary

**Figure:**
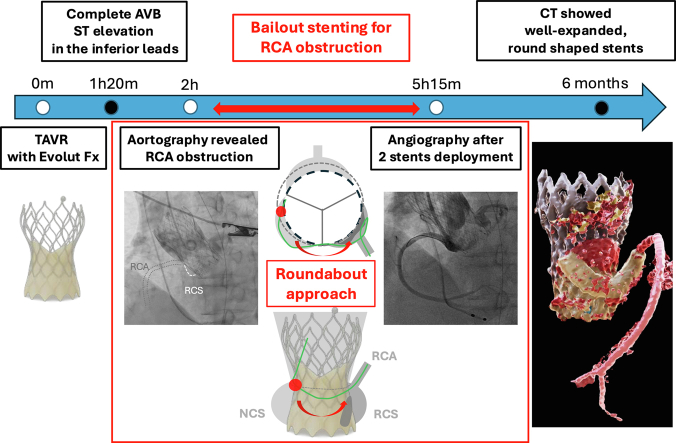

**Visual Summary:**
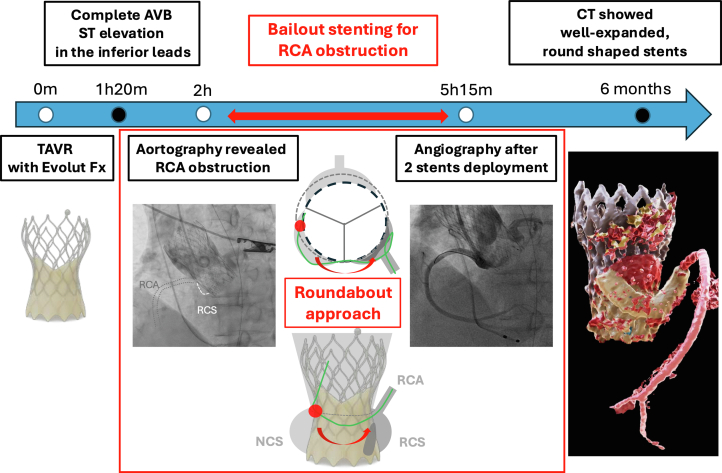
Timeline of Case Presentation AVB = atrioventricular block; CT = computed tomography; RCA = right coronary artery; TAVR = transcatheter aortic valve replacement.

Coronary artery obstruction (CO) is an uncommon but potentially fatal complication of transcatheter aortic valve replacement (TAVR). Based on limited published data, CO occurs more frequently with balloon-expandable valves and most commonly affects the left coronary artery (LCA).^1^ Although bailout percutaneous coronary intervention (PCI) is essential when CO occurs, it can be technically challenging and may sometimes require conversion to open heart surgery. Selective engagement and revascularization of the right coronary artery (RCA) after implantation of self-expanding valves (SEVs) can be particularly difficult, even in elective cases.^2^

**Take-Home Message**

• In bailout PCI for TAVR-related coronary obstruction, particularly when sequestration of the coronary sinus prevents direct cannulation, a “roundabout” approach via the adjacent coronary cusp can facilitate coronary access and successful revascularization.

Here, we describe a case of subacute RCA obstruction after TAVR with SEV—arguably one of the most demanding scenarios for bailout PCI—in which revascularization was successfully achieved using a “roundabout” approach that accessed the right coronary sinus (RCS) via the noncoronary sinus (NCS).

## History of Presentation

**Figure 1:**
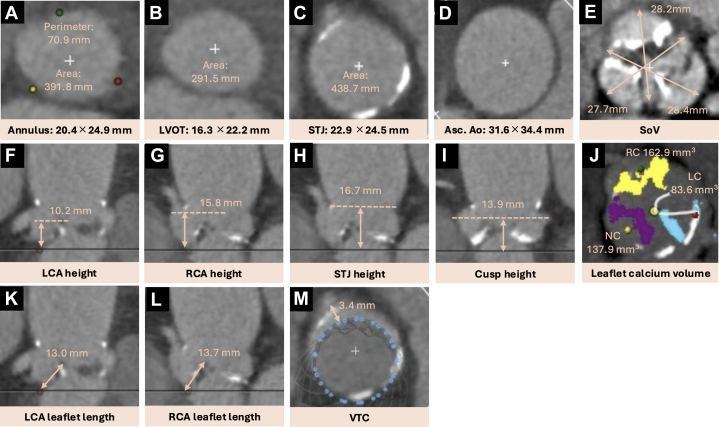
Preprocedural Computed Tomography Measurements (A to E) The anatomical features of the aortic valve were deemed suitable for implantation of a 26-mm Evolut FX valve. The overall risk of coronary artery obstruction was estimated to be low based on several parameters: (E) SoV diameters, (G) RCA height, (I) cusp height, and (J) calcium volume of the right coronary leaflet. However, (M) the VTC distance was measured at 3.4 mm, which was the only parameter below the established safety cutoff. Asc. Ao = ascending aorta; LC = left coronary; LCA = left coronary artery; LVOT = left ventricular outflow tract; NC = noncoronary; RC = right coronary; RCA = right coronary artery; SoV = sinus of Valsalva; STJ = sinotubular junction; VTC = valve-to-coronary.

**Figure 2:**
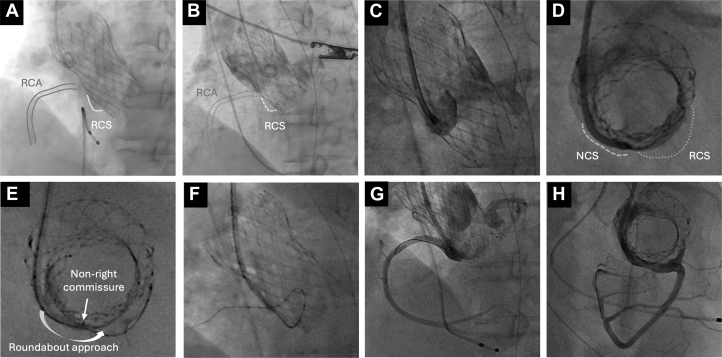
Angiography During Transcatheter Aortic Valve Replacement and Percutaneous Coronary Intervention (A) Immediately after transcatheter aortic valve replacement, aortic angiography demonstrated opacification of both the RCA and RCS. (B) However, angiography after the patient's sudden deterioration failed to visualize either structure. (C and D) Selective contrast injection from the NCS via a guiding catheter partially opacified the RCS. (E and F) Based on this finding, a guidewire was introduced through the NCS, successfully crossing the RCA. (G and H) Final coronary angiography showed TIMI flow grade 3. NCS = noncoronary sinus; RCA = right coronary artery; RCS = right coronary sinus.

A 91-year-old woman with symptomatic severe aortic stenosis was referred to our hospital for TAVR. During the preoperative evaluation, allergy to cobalt/chromium was identified. Accordingly, a 26-mm Evolut FX valve (Medtronic), which was free of these metals, was selected based on preprocedural computed tomography (CT) assessment (Figure 1). The TAVR procedure was completed without major complications, and postprocedural aortic angiography demonstrated preserved coronary flow (Figure 2A); however, minimal space was observed between the transcatheter heart valve (THV) frame and the aortic wall at the RCS near the sinotubular junction (STJ) (Video 1). Shortly after transfer to the intensive care unit, the patient developed complete atrioventricular block followed by cardiopulmonary arrest. After return of spontaneous circulation, electrocardiography showed ST-segment elevation in leads II, III, and aVF.

## Past Medical History

The patient was taking medication for hypertension, dyslipidemia, and Hashimoto's thyroiditis.

## Differential Diagnosis

Complete atrioventricular block is a relatively common complication after TAVR, particularly with SEVs. In this case, an isolated complete atrioventricular block was initially suspected; however, concomitant ST-segment elevation in the inferior leads on the electrocardiogram strongly suggested a subacute RCA compromise. Differential considerations for subacute CO after TAVR typically include delayed THV expansion, coronary embolization, THV displacement, aortic root dissection, and thrombus formation.^1^^,^^3^^,^^4^

## Investigations

Preprocedural CT revealed an aortic annulus area of 391.8 mm^2^ and a perimeter of 70.9 mm. The STJ measured 22.9 × 24.5 mm in diameter, with a height of 16.7 mm. The diameters of the sinuses of Valsalva (SoV) were 28.4 mm (left), 28.2 mm (right), and 27.7 mm (noncoronary). The heights of the LCA and RCA were 10.2 mm and 15.8 mm, respectively. The calcium volume of the right coronary leaflet was 162.9 mm^3^. The valve-to-coronary distance measured 3.4 mm (Figure 1). The SoV diameters and short valve-to-coronary distance indicated a potential risk of CO. However, given the sufficient height of the RCA, coronary protection during TAVR was not considered mandatory. Preprocedural coronary angiography revealed no significant stenosis in the coronary arteries. Right heart catheterization showed a mean pulmonary capillary wedge pressure of 10 mm Hg and a cardiac index of 2.8 L/min/m^2^, indicating hemodynamic stability.

The 26-mm Evolut FX valve was implanted at the depth of 2 mm below the aortic annulus. Postprocedural aortography revealed preserved flow in the coronary arteries (Figure 2A, Video 1).

## Management

**Figure 3:**
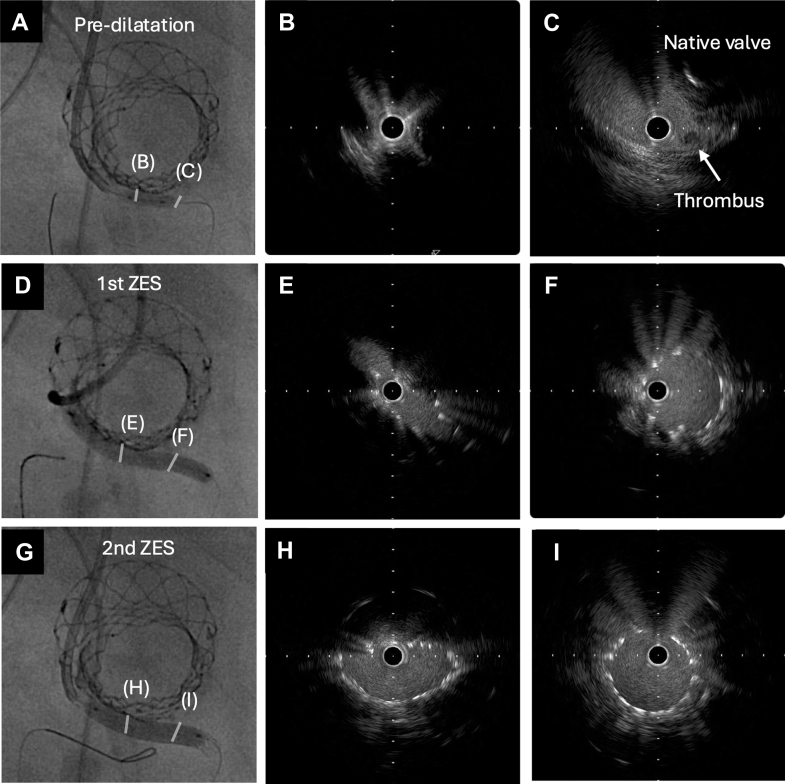
Intravascular Ultrasound Before and After Stent Implantation (A to C) IVUS demonstrating the presence of a thrombus and native valve tissue within the right coronary sinus. (D to F) After implantation of the first zotarolimus-eluting stent (ZES), IVUS showed compression of the stent by the transcatheter heart valve. (G to I) Subsequent IVUS imaging after the placement of a second overlapping ZES and postdilation with a 5.0-mm noncompliant balloon confirmed the restoration of an acceptable luminal area. IVUS = intravascular ultrasound.

**Figure 4:**
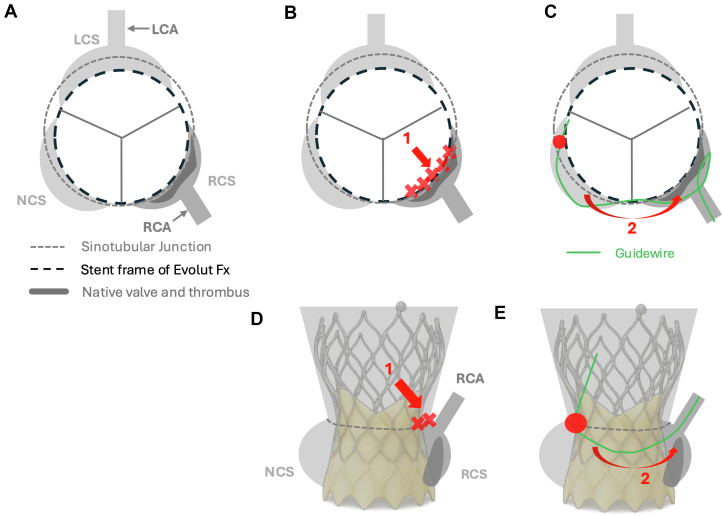
Schematic Representation of the Ipsilateral and Roundabout Approaches (A) Sequestration of the RCS leads to reduced sinus flow and thrombus formation. Red arrows in (B) to (E) indicate access routes: arrow 1 represents the ipsilateral approach and arrow 2 represents the roundabout approach. (B and D) The ipsilateral approach failed because of obstruction by the transcatheter heart valve and native aortic valve leaflets, which prevented coronary access. (C and E) The roundabout approach, tracking along the outer frame of the transcatheter heart valve from the NCS to the RCS, successfully enabled guidewire crossing into the RCA. LCA = left coronary artery; LCS = left coronary sinus; NCS = noncoronary sinus; RCA = right coronary artery; RCS = right coronary sinus.

Shortly after return of spontaneous circulation, the patient was brought to the catheterization laboratory, and a temporary pacemaker was inserted via the right internal jugular vein. As aortography suggested an RCA obstruction (Figure 2B, Video 2), PCI to the RCA was performed immediately. To preserve femoral access for mechanical circulatory support in case of hemodynamic collapse, a 6/7-F glidesheath was placed in the left radial artery and a 6-F JR3.5 guiding catheter was advanced; however, initial attempts to cross the lesion using a guidewire supported by an extension catheter and microcatheter were unsuccessful. We then switched to a 6-F Mach 1 MP guiding catheter (Boston Scientific) and reattempted the guidewire crossing. Selective contrast injection from the NCS partially opacified the RCS. Based on this finding, we successfully advanced the guidewire assisting an extension catheter and a microcatheter into the RCA via a “roundabout” approach, crossing over the NCS to the non-right commissure (Figures 2C to 2F). Intravascular ultrasound confirmed the thrombus and native leaflet tissue within the RCS (Figures 3A to 3C, Video 3). After balloon expansion, a zotarolimus-eluting stent was implanted. However, intravascular ultrasound revealed significant deformation (minimal lumen area: 3.2 mm^2^, ellipticity index: 0.44). Consequently, a second overlapping stent was added to enhance radial strength, which successfully improved expansion (minimal lumen area: 10.5 mm^2^, ellipticity index: 0.47) (Figures 3D to 3I, Video 4). Final imaging confirmed satisfactory stent expansion and coronary flow restoration (Figures 2G and 2H). A schematic of this strategy is shown in Figure 4.

## Outcome and Follow-Up

**Figure 5:**
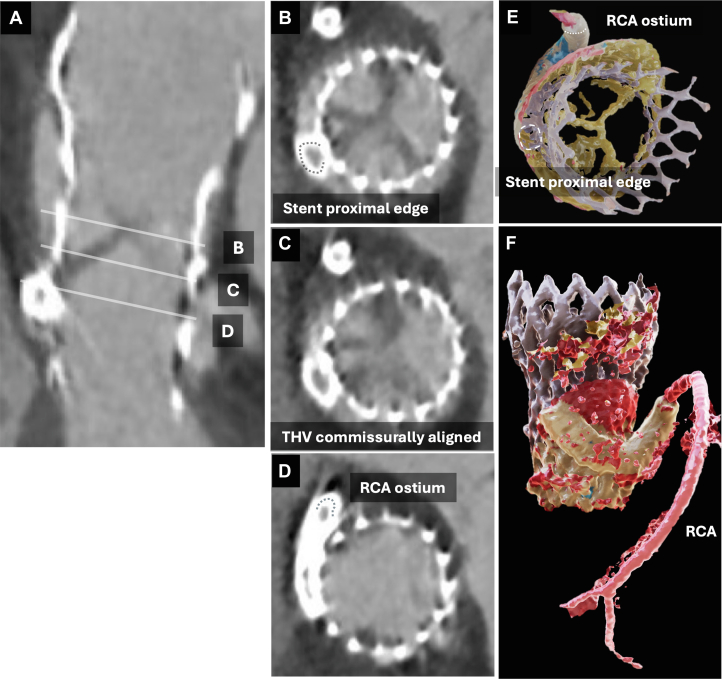
Computed Tomography and 3-Dimensional Reconstruction Images at 6-Month Follow-Up (A to D) At 6 months, the stents were positioned at the proximal RCA via a route from the NCS through the transcatheter heart valve strut above the RCS. (B to E) Detailed computed tomography assessment demonstrates that commissural alignment was successfully achieved and considered favorable. (E and F) The roundabout approach is clearly visualized through the 3-dimensionally reconstructed images (reconstructed using Viewtify, Sciement). Abbreviations as in Figure 4.

The patient was extubated on postoperative day 2. The peak creatine kinase level was 1,956 U/L. After initiating cardiac rehabilitation, she was discharged on postoperative day 21 without any disabilities. Follow-up CT at 6 months demonstrated patent stents with no evidence of recoil or restenosis (Figure 5). Given the absence of any bleeding events, dual antiplatelet therapy was continued.

## Discussion

CO after TAVR is rare, occurring in <1% of cases, but it is associated with substantial mortality. Evidence on CO after TAVR is limited, and optimal bailout strategies have not yet been established. To the best of our knowledge, this is the first report of PCI using the “roundabout” approach for subacute RCA occlusion after SEV implantation.

Several registries have reported the incidence and potential risk factors for CO. It occurs more frequently in women and older patients, and it is more common with balloon-expandable valves. Approximately 90% of cases involve the LCA.^1^^,^^3^^,^^4^ Reported CT predictors of CO include low coronary ostium height, small SoV, bulky calcification, increased leaflet length, a virtual valve-to-coronary distance of ≤4 mm, and valve-in-valve procedures.^1^^,^^3^^,^^4^ Recent multivariate models incorporating cusp height greater than ostium height, combined with a valve-to-coronary distance of ≤4 mm or culprit leaflet calcium volume of >600 mm^3^, have demonstrated strong predictive performance for CO, with areas under the curve of 0.93 for the LCA and 0.94 for the RCA.^5^ Additionally, when the annular diameter exceeds the STJ diameter and the cusp height is greater than the STJ height, the leaflet may protrude above the STJ and sequester the sinus, potentially impeding coronary flow.^6^ Several established risk factors were identified for our patient.

Recently, a case series reported that calcification at the STJ level was displaced toward the RCA by a THV, resulting in compression and subsequent occlusion of an upwardly coursing RCA.^7^ In our case, although the RCA coursed upward and there was mild calcification at the STJ level, this mechanism was considered unlikely because no evidence of external compression was seen on intravascular ultrasound.

CO can be broadly classified into 3 categories based on the timing of its onset. *Acute CO*, occurring intraprocedurally, is often associated with direct obstruction.^6^ *Delayed CO* (DCO) is defined as CO occurring after a patient leaves the operating room, and it is further subdivided into 2 categories. *Early DCO*, which occurs within 7 days of the procedure, has been associated with mechanisms such as delayed expansion of the THV, development of a dissection flap, and thrombus formation.^4^^,^^8^ *Late DCO*, occurring >7 days postimplantation, is often attributed to THV endothelialization and thrombus formation around the displaced native leaflets, leading to progressive sinus isolation.^6^ In our case, shallow SEV implantation within a low SoV height was considered the primary cause of RCS sequestration, which subsequently led to blood flow stagnation and thrombus formation. Therefore, especially in cases with low SoV, avoiding shallow implantation is crucial to minimize the risk of sinus sequestration.

We performed bailout PCI in a rare case of subacute RCA occlusion after SEV implantation. Bailout PCI has a failure rate of 20% to 30% regardless of the timing of CO, primarily because of difficulties in coronary access caused by interference from THV frames or native valve leaflets.^1^^,^^2^ In 66.6% of failed PCI cases, failure was due to unsuccessful coronary cannulation or guidewire crossing.^1^ RCA cannulation after SEV placement is known to be challenging, even during elective PCI after TAVR. In our case, cannulation from the culprit sinus was virtually impossible because the RCS was isolated from the THV skirt. A previous report described accessing the left coronary sinus via the NCS using a “roundabout” approach in a case of late DCO.^9^ Our case demonstrates that the same strategy for RCA access can be used in an emergency. This roundabout technique offers an alternative route when sinus sequestration precludes direct coronary access. After single stent deployment, intravascular ultrasound showed deformation caused by THV compression. A second overlapping stent was added to enhance radial strength,^10^ effectively improving lumen area and symmetry. As reported,^9^ multiple overlapping stents may be essential to withstand such compressive forces and ensure adequate vessel patency.

Finally, although midterm patency was confirmed in our patient, the long-term durability of this approach remains uncertain and further investigation is needed.

## Conclusions

When CO is caused by sinus sequestration after self-expanding TAVR, a “roundabout” bailout strategy via the noncoronary cusp can facilitate RCA access and successful revascularization if direct cannulation is not feasible.

## Funding Support and Author Disclosures

The authors have reported that they have no relationships relevant to the contents of this paper to disclose.

## Appendix

Video 1 A 26-mm Evolut FX Valve Was Implanted at a High Position However, the right coronary artery occluded soon after the transfer to the intensive care unit (see Video 2).

Video 2 Right Coronary Artery Occlusion Soon After the Patient Was Transferred to the Intensive Care Unit

Video 3 Sequential Intravascular Ultrasound Images Before Stenting For intravascular ultrasound images after second overlapping stent deployment, see Video 4.

Video 4 Sequential Intravascular Ultrasound Images After Second Overlapping Stent Deployment
